# Regularization of electromagnetic scattering problems via the Abel integral transform

**DOI:** 10.1098/rsta.2024.0349

**Published:** 2025-08-14

**Authors:** Elena Vinogradova, Paul Smith

**Affiliations:** ^1^ School of Mathematical and Physical Sciences, Macquarie University, Sydney, New South Wales, Australia

**Keywords:** Abel integral transform, method of analytical regularization, electromagnetic wave scattering, integral equations, open shells with slots and apertures, dual series equations

## Abstract

The Abel integral transform is a powerful mathematical tool for solving mixed boundary value problems for the Helmholtz and Maxwell equations. It is particularly effective for treating two- and three-dimensional electromagnetic wave scattering from cavity backed apertures. Such scattering problems give rise to dual, triple (and higher order) series and integral equations. These equations are inherently ill-posed and discretization results in ill-conditioned systems that resist stable numerical solution. Their regularization commences by representing the basis functions occurring in the equations in terms of Jacobi polynomials of a particular class. A sequence of Abel integral transforms is applied to each member of the series equations producing Jacobi polynomials of a different class. The transforms are arranged so that the resulting system is well-posed and may be converted to a well-conditioned Fredholm matrix system of second kind. Its numerical solution provides stable and convergent results of guaranteed accuracy. This paper discusses the treatment of three typical examples of dual and triple series arising in electromagnetic wave scattering from ideally conducting arbitrary slotted cylinders and axisymmetric thin-walled shells with one or two apertures. These are among the examples most commonly encountered in scattering problems of this nature.

This article is part of the theme issue ‘Analytically grounded full-wave methods for advances in computational electromagnetics’.

## 1. Introduction

Regularization is an important, often essential, step in guaranteeing the convergence of numerical methods applied to the various formulations of electromagnetic (EM) scattering problems. Many canonical geometries (such as spheres or cylinders of circular or elliptical cross section) or structures with other special features are amenable to treatments which result in stable calculational procedures possessing guaranteed accuracy. However, problems of greater generality, such as scattering from arbitrarily shaped conducting obstacles with one or more apertures, cannot take advantage of such special treatments. Such problems are usually formulated in terms of an integral equation (IE), or a system of IEs, utilizing a relevant Green’s function with a singular (or hyper-singular) kernel; the IE is usually a Fredholm integral equation of first kind, a class of equations that is recognized to be ill-posed and numerical discretization produces systems that are ill-conditioned and unstable. The ill-posedness arises from the fact that, although the solution to the IE exists and is unique, it does not depend continuously on the input data (namely, the incident field). By contrast, a well-posed problem, in the sense defined by Jacques Hadamard, is one for which a solution exists, is unique and depends continuously on the input data. Such features are highly desirable in any mathematical model of a physical process. While numerical regularization techniques (such as Tikhonov regularization) can be considered, regularization of the underlying equations by analytical means is preferable in producing a system of equations that is equivalent to a well-posed Fredholm IE of second kind, the great advantage being that standard numerical algorithms are easily applied to produce well-conditioned systems that guarantee stable and convergent results.

The purpose of this paper is of a review nature. It describes the Abel integral transform method and surveys its application in a variety of scattering problems in two dimensions and for three-dimensional bodies of revolution. Its attractiveness lies in its wide applicability, not relying on special features of the underlying geometry or other parameters of the problem. The analytically regularized set of equations is a matrix system (of infinite order) of second kind, and standard truncation methods yield numerical solutions whose convergence to the exact solution is assured, as the truncation order is increased without limit. Solutions produced in this way may justifiably be described as ‘benchmarks’, against which solutions generated from other scattering formulations can be compared, particularly where the stability or convergence of the numerical methods employed cannot be rigorously established.

The so-called Method of Analytical Regularization (MAR) encompasses a variety of approaches aimed at the transformation of the boundary value problems (BVPs) in wave scattering to Fredholm second kind infinite-matrix equations. Detailed descriptions of various implementations of the MAR may be found in many sources (e.g. [1–6]). Briefly, the common idea in all implementations may be formally described as follows.

Its key idea is a regularization procedure, with the aim of converting the ill-posed equations of the first kind (which arise in the initial formulation of the scattering problem) into well-conditioned equations of the second kind. From a technical point of view, the first kind equations may initially be expressed as Ax=b, where A,x and b denote the operator relevant to the problem, the unknown function to be found, and the known excitation, respectively. The operator A is split into singular and regular parts $A_{0}$ and $A_{1}$ so that $\left(A_{0}+A_{1}\right)x=b$. In some situations, $A_{0}$ corresponds to the static part of the full-wave problem. The inverse operator $A_{0}^{-1}$ is constructed analytically and applied to the equation giving $\left(I+H\right)x=\tilde{b}$, where $I=A_{0}^{-1}A_{0}$ is the identity operator, $H=A_{0}^{-1}A_{1}$ and $\tilde{b}=A_{0}^{-1}b$. Although the decomposition of $A_{0}$ may be done in a variety of ways, it must be chosen that the operator H is a completely continuous (or compact) operator in the space $l_{2}$ of square summable sequences—thus making the transformed or regularized equation of second kind Fredholm type; also the modified excitation $\tilde{b}$ should belong to $l_{2}$.

Abel’s integral transform is pivotal in the analytical construction of the inverse operator $A_{0}^{-1}$. To understand the background, the rigorous statement of problems concerning acoustic and electromagnetic wave diffraction from open shells or open cylinders leads to a set of dual (or triple or multiple) series equations or dual (or triple or multiple) integral equations to be solved. Although there is no comprehensive theory for solving such equations, three basic approaches in various treatments can be discerned: the definition-extension method [7], the substitution method [8] and the multiplying factor method [9]. Their key steps are reviewed in [4]: their common distinctive feature is the utilization, in one form or another, of Abel’s integral equation (or transform) to produce a matrix system of second kind.

The Abel transform technique was not the first implementation of the MAR in electromagnetics. The first seems to have occurred in about 1962 in the study [10] of two-dimensional scattering from perfectly electrically conducting (PEC) flat or curved strips of zero-thickness. Drawing on the theory of analytical functions, the known analytical solution to the classical Riemann–Hilbert problem was used to effect a semi-inversion of the full-wave problem (see also [11]). This approach was later extended to two-dimensional scattering from PEC circular screens [12–14], non-circular smooth PEC screens [15,16], impedance surfaces [17] and other non-PEC structures. This method for the solution of BVPs in electromagnetics is comprehensively described in [1] and is further highlighted in the review [3]. Its use is restricted to two-dimensional EM scattering problems: the problems soluble by this method are equally soluble by the Abel transform method and the results are identical. Since the Abel transform method is also applicable to certain three-dimensional scattering problems, it might be viewed as a possibly more universal tool for this purpose.

A second technique, the Regularizing Galerkin Technique or Method of Analytical Preconditioning, also appeared in the early 1960s [18,19]. The technique is applied to the electric-field integral equation (EFIE) for the current induced on a zero-thickness PEC strip. It uses weighted Chebyshev polynomials (of first kind in the E-polarization case and second kind in H-polarization) as a full-wave expansion basis; these polynomials are the orthogonal eigenfunctions of the static part of the EFIE operator in the respective polarizations. This Galerkin projection scheme combines the discretization of the EFIE with analytical inversion of the singular part of the integral operator; the treatment of the remaining smooth part is straightforwardly incorporated. The technique can be modified to treat the scattering from two-dimensional polygonal scatterers (i.e. non-smooth surfaces) by replacing the Chebyshev polynomials with weighted Jacobi polynomials with parameters determined by the edge angles. PEC polygonal cylinders were studied in [20] and dielectric polygonal cylinders in [21]. The set of Jacobi polynomials, as a full-wave basis, appears also in the three-dimensional scattering from a PEC circular hollow pipe [22] and disc [23].

This article focuses on the role of the Abel integral transform method as a tool with wide generality and applicability. It is particularly effective in solving problems concerning scattering from cavity backed apertures. A formulation with single- or double-layer potentials gives rise to dual (or triple) series to which the method will be applied. Three types of structures are chosen to illustrate the aspects most commonly encountered. The next two sections describe Abel’s integral equation and Abel-type integral representations of the functions usually arising in two- and three-dimensional scattering. In §4, the basic idea of the approach is illustrated with possibly the simplest example (a sphere with a circular aperture). The next two sections discuss more sophisticated applications: §5 discusses two-dimensional EM wave scattering problems for slotted cylinders of arbitrary profile, whereas §6 considers EM scattering by three-dimensional open shells possessing rotational symmetry. Further prospects for utilization of the Abel integral transform method are discussed.

## 2. Abel’s integral equation and Abel integral transforms

Abel’s integral equation is sometimes considered as a precursor of the modern theory of linear integral equations. Originally, the integral equation


(2.1) $$f\left(x\right)=\int_{0}^{x}\frac{u\left(\xi \right)d\xi }{\sqrt{x-\xi }},$$

where f is a known function and u is the function to be determined, arose from some problem in mechanics. Instead of equation (2.1), Abel set himself the problem of solving the more general equation


(2.2) $$f\left(x\right)=\int_{a}^{x}\frac{u\left(\xi \right)d\xi }{{\left(x-\xi \right)}^{\lambda }}\;\;\;\;\;\;\;\;\;\;\;\;\;\;\left(a<x<b\right),$$

where the parameters a,b (a<b) and λ are fixed, with 0<λ<1. Details of its solution can be found in several texts, including [24–26]; we simply state the inversion formula for (2.2):


(2.3) $$u\left(\xi \right)=\frac{\sin\lambda \pi }{\pi }\frac{d}{d\xi }\int_{a}^{\xi }\frac{f\left(x\right)dx}{{\left(\xi -x\right)}^{1-\lambda }}.$$

The companion form of this generalized Abel integral equation,


(2.4) $$f\left(x\right)=\int_{x}^{b}\frac{u\left(\xi \right)d\xi }{{\left(\xi -x\right)}^{\lambda }}\;,\;\;\;\;\;\;\;\;\;\;\;\;\;\left(a<x<b\right)$$

has corresponding inversion formula


(2.5) $$u\left(\xi \right)=-\frac{\sin\lambda \pi }{\pi }\frac{d}{d\xi }\int_{\xi }^{b}\frac{f\left(x\right)dx}{{\left(x-\xi \right)}^{1-\lambda }}.$$

The conditions under which the inversion formulae (2.3) and (2.5) are valid are discussed in [27]. The particular result that we will repeatedly use in this paper is the following.

**Theorem**. *The unique continuous solution* u *on* $\left(a,b\right)$ *to the homogeneous form of the integral equation (2.2), in which* $f\left(x\right)\equiv 0,$ *is the zero solution* $u\left(x\right)\equiv 0$. *The same result holds for integral equation (2.4*).

The pair (2.2) and (2.3) may be considered as companion integral transforms. If we regard the transform (2.2) as the direct Abel integral transform (of the function *u*), then integral transform (2.3) is its inverse. Similar terminology can be applied to the pair (2.4) and (2.5).

When solving acoustic and EM scattering problems for open cylinders of arbitrary cross section (in two dimensions) and open shells (in three dimensions), dual (or triple and higher order) series equations are formed after implementation of the relevant mixed boundary value conditions. The usual basis functions employed in this process are the exponential functions $e^{in\phi }$ (n integer) along with the associated Legendre polynomials $P_{n}^{m}\left(\cos\theta \right)$ (n,m integers) with trigonometric argument. Abel’s integral transform is more conveniently applied with the trigonometric functions cos⁡nϕ and sin⁡nϕ rather than the exponential function $e^{in\phi }=\cos n\phi +i\sin n\phi$. These basis functions can be expressed in terms of Jacobi polynomials $P_{n}^{\left(\alpha ,\beta \right)}\left(x\right)$ of suitable degree n and appropriately chosen parameters α>−1,β>−1. As is well known, for fixed α,β,the set of Jacobi polynomials is orthogonal on the interval $\left(-1,1\right)$ with weight function $w\left(x\right)={\left(1-x\right)}^{\alpha }{\left(1+x\right)}^{\beta }$; it provides a complete basis for the Hilbert space $L_{w}^{2}\left[-1,1\right]$ of functions that are square integrable with weight w on $\left[-1,1\right]$. The regularization technique expounded in this review relies upon representing the Jacobi polynomial basis $P_{n}^{\left(\alpha ,\beta \right)}\left(x\right)(n=0,1,2,\ldots )$ as Abel integral transforms of Jacobi polynomials $P_{n}^{\left(\alpha^{\prime },\beta^{\prime }\right)}\left(x\right)\;\left(n=0,1,2,\;\ldots \right)$ with different parameters $\alpha^{\prime }>-1,\beta^{\prime }>-1$. In each of the dual (or triple and higher order) series members these parameters are chosen to ensure that the final regularized system of equations is of second kind. The next section outlines these representations.

## 3. Abel-type integral representations

Many hypergeometric functions have Abel-type integral representations; an expanded list can be found in [4]. We focus on the representations of those frequently occurring functions encountered in solving practical EM wave scattering problems, namely, the trigonometric functions and the associated Legendre polynomials. The trigonometric functions of integer or half-integer order are connected to the Jacobi polynomials as follows:


(3.1) $$\cos n\theta =\sqrt{\pi }\frac{\Gamma \left(n+1\right)}{\Gamma \left(n+\frac{1}{2}\right)}P_{n}^{\left(-\frac{1}{2},-\frac{1}{2}\right)}\left(\cos\theta \right),$$

(3.2) $$\cos\left(n+\frac{1}{2}\right)\theta =\sqrt{\pi }\frac{\Gamma \left(n+1\right)}{\Gamma \left(n+\frac{1}{2}\right)}{\cos\frac{1}{2}\theta P}_{n}^{\left(-\frac{1}{2},\frac{1}{2}\right)}\left(\cos\theta \right),$$

(3.3) $$\sin\mathrm{n\theta}=\frac{\sqrt{\pi }}{2}\frac{\Gamma \left(n+1\right)}{\Gamma \left(n+\frac{1}{2}\right)}{\sin\theta P}_{n-1}^{\left(\frac{1}{2},\frac{1}{2}\right)}\left(\cos\theta \right),$$

(3.4) $$\sin\left(n+\frac{1}{2}\right)\theta =\sqrt{\pi }\frac{\Gamma \left(n+1\right)}{\Gamma \left(n+\frac{1}{2}\right)}\sin\frac{1}{2}\theta \cdot P_{n}^{\left(\frac{1}{2},-\frac{1}{2}\right)}\left(\cos\theta \right).$$

For the associated Legendre polynomials, the connection is


(3.5) $$P_{n}^{m}\left(\cos\theta \right)=2^{-m}{\left(\sin\theta \right)}^{m}\cdot \frac{\Gamma \left(n+m+1\right)}{\Gamma \left(n+1\right)}P_{n-m}^{\left(m,m\right)}\left(\cos\theta \right).$$

The Abel-type integral representation of the Jacobi polynomial $P_{n}^{\left(\alpha ,\beta \right)}\left(x\right)$ of degree n in terms of the Jacobi polynomial $P_{n}^{\left(\alpha^{`},\beta^{`}\right)}\left(x\right)$ of the same degree but different parameters $\alpha^{\prime },\beta^{\prime }$ is established in [4]. Let the parameter η be chosen to lie in the interval 0≤η<1. Then if α+η>0,


(3.6) $$P_{n}^{\left(\alpha ,\beta \right)}\left(t\right)=\frac{{\left(1-t\right)}^{-\alpha }\Gamma \left(n+1+\alpha \right)}{\Gamma \left(1-\eta \right)\Gamma \left(n+\alpha +\eta \right)}\int_{t}^{1}\frac{{\left(1-x\right)}^{\alpha +\eta -1}P_{n}^{\left(\alpha +\eta -1,\beta -\eta +1\right)}\left(x\right)}{{\left(x-t\right)}^{\eta }}dx,$$

while if β+η>0,


(3.7) $$P_{n}^{\left(\alpha ,\beta \right)}\left(t\right)=\frac{{\left(1+t\right)}^{-\beta }\Gamma \left(n+1+\beta \right)}{\Gamma \left(1-\eta \right)\Gamma \left(n+\beta +\eta \right)}\int_{-1}^{t}\frac{{\left(1+x\right)}^{\beta +\eta -1}P_{n}^{\left(\alpha -\eta +1,\beta +\eta -1\right)}\left(x\right)}{{\left(t-x\right)}^{\eta }}dx.$$

Simple rearrangement of formulae (3.6) and (3.7) shows that


$${\left(1-t\right)}^{\alpha }P_{n}^{\left(\alpha ,\beta \right)}\left(t\right)/\Gamma \left(n+1+\alpha \right)\;$$

is an Abel integral transform of


$$\Gamma {\left(1-\eta \right)}^{-1}\cdot {\left(1-x\right)}^{\alpha +\eta -1}P_{n}^{\left(\alpha +\eta -1,\beta -\eta +1\right)}\left(x\right)/\Gamma \left(n+\alpha +\eta \right),$$

and


$$(1+t{)}^{\beta }P_{n}^{\left(\alpha ,\beta \right)}(t)/\Gamma \left(n+1+\beta \right)$$

is an Abel integral transform of


$$\Gamma {\left(1-\eta \right)}^{-1}\cdot {\left(1+x\right)}^{\beta +\eta -1}P_{n}^{\left(\alpha -\eta +1,\beta +\eta -1\right)}\left(x\right)/\Gamma \left(n+\beta +\eta \right),$$

on the appropriate intervals. Formulae (3.6) and (3.7) also have an interpretation in terms of fractional integration operators [13]. When η=0, two notable identities corresponding to integration in the conventional sense result:


(3.8) $${\left(1-t\right)}^{\alpha +1}P_{n}^{\left(\alpha +1,\beta -1\right)}\left(t\right)=\left(n+\alpha +1\right)\int_{t}^{1}{\left(1-x\right)}^{\alpha }P_{n}^{\left(\alpha ,\beta \right)}\left(x\right)dx$$

(3.9) $${\left(1+t\right)}^{\beta +1}P_{n}^{\left(\alpha -1,\beta +1\right)}\left(t\right)=\left(n+\beta +1\right)\int_{-1}^{t}{\left(1+x\right)}^{\beta }P_{n}^{\left(\alpha ,\beta \right)}\left(x\right)dx.$$

Two further relationships among the Jacobi polynomials are important for the solution of the dual (triple) series equations involving Jacobi polynomials. The first is *Rodrigues’ formula* [28]:


(3.10) $$-n{\left(1-x\right)}^{\alpha }{\left(1+x\right)}^{\beta }P_{n}^{\left(\alpha ,\beta \right)}\left(x\right)=\frac{d}{dx}\left\{{\left(1-x\right)}^{\alpha +1}{\left(1+x\right)}^{\beta +1}P_{n}^{\left(\alpha +1,\beta -1\right)}\left(x\right)\right\}.$$

The second expresses the ultraspherical polynomials of even or odd degree in terms of a Jacobi polynomial [29]:


(3.11) $$P_{2n+l}^{\left(\alpha ,\alpha \right)}\left(z\right)=\frac{\Gamma \left(n+1\right)}{\Gamma \left(2n+1+l\right)}\cdot \frac{\Gamma \left(2n+\alpha +1+l\right)}{\Gamma \left(n+\alpha +1\right)}z^{l}P_{n}^{\left(\alpha ,l-\frac{1}{2}\right)}\left(2z^{2}-1\right),\;\;\left(l=0,1\right).$$

This relationship (3.11) is the key to the transformation of symmetrical triple equations with Jacobi polynomials into two independent dual series equations [4]: for Legendre polynomials (3.11) gives


(3.12) $$P_{2n+l}\left(\cos\theta \right)={\left(\cos\theta \right)}^{l}P_{n}^{\left(0,l-\frac{1}{2}\right)}\left(\cos2\theta \right).$$

## 4. Abel’s integral transform method: a simple example

While it has much in common with the use of fractional integration [30] for solving dual integral equations, Abel’s integral transform method may be illustrated with a simple example, the determination of the electrostatic potential of a charged PEC spherical cap of angular size $\theta_{0}$ when its surface is held at a constant potential of unit value. A precise statement of the problem is as follows. It is required to find the potential U satisfying the conditions (i) ΔU=0 at all points p of space except on the cap, (ii) U is continuous everywhere (off and on the cap) and takes unit values at all points on the cap, (iii) the normal (or radial) derivative of U is continuous across the aperture, (iv) U=U(p)→0 as |p|→∞, and (v) the energy $\overset{}{\underset{V}{∭}}{\left|\nabla U\right|}^{2}dV$ in any volume *V* including the sharp edges is finite.

This problem produces the following dual series equations involving the Legendre polynomials (see (3.5)) $P_{n}\left(\cos\theta \right)\equiv P_{n}^{\left(0,0\right)}\left(\cos\theta \right)$:


(4.1) $$\sum_{n=0}^{\infty }\left(2n+1\right)a_{n}P_{n}^{\left(0,0\right)}\left(x\right)=0,x\in \left(-1,x_{0}\right)$$

(4.2) $$\sum_{n=0}^{\infty }a_{n}P_{n}^{\left(0,0\right)}\left(x\right)=1,\;\;\;\;\;\;\;\;\;\;\;\;\;\;\;\;\;\;\;\;\;x\in \left(x_{0},1\right),$$

where $x=\cos\theta ,x_{0}=\cos\theta_{0}$. The condition of boundedness of the electrostatic energy U in any arbitrary volume V including the edges,


(4.3) $$\overset{}{\underset{V}{∭}}{\left|\nabla U\right|}^{2}dV<\infty ,$$

imposes the constraint


(4.4) $$\sum_{n=0}^{\infty }\frac{n+1}{2n+1}{\left|a_{n}\right|}^{2}<\infty$$

on the sequence of unknown coefficients ${\left\{a_{n}\right\}}_{n=0}^{\infty }$, thus requiring it to belong to the Hilbert space $l_{2}$. The Legendre polynomials have the asymptotic estimate $P_{n}^{\left(0,0\right)}\left(x\right)=O\left(n^{-1/2}\right)$ as n→∞. The processes in this section employ operations such as term-by-term integration or the interchange of the order of integration and summation for equations (4.1) and (4.2); the same processes have their counterparts in subsequent sections. A rigorous justification for these operations is provided in [4, ch. 2].

The main role of the Abel integral transform is to convert the dual series (4.1) and (4.2)—which are defined on the adjoining subintervals $\left(-1,x_{0}\right)$ and $\left(x_{0},1\right)$ of the full interval $\left(-1,1\right)$—into an equation of the form


(4.5) $$F\left(x\right)=\left\{ \begin{array}{l} F_{1}\left(x\right),\;\;\;\;\;\;\;x\in \left(-1,x_{0}\right)\\ F_{2}\left(x\right),\;\;\;\;\;\;\;\;\;\;x\in \left(x_{0},1\right) \end{array}\right.$$

for a suitably defined unique function $F\left(x\right)$, which is piecewise continuous in the full interval, and functions $F_{1}\left(x\right),F_{2}\left(x\right)$ obtained by the corresponding transformations of the right-hand sides of (4.1) and (4.2).

The strategy applies a suitable Abel’s integral transform to (4.1) so that in the new polynomial basis the factor (2n+1) is replaced by a factor which is $O(n^{\frac{1}{2}})$ as n→∞, while a suitable inverse transform applied to (4.2) inserts a factor (in the same new polynomial basis) which is also $O(n^{\frac{1}{2}})$ as n→∞. The resultant dual series equations employ a polynomial basis in which the rate of decay of the coefficients is the same for the corresponding members of the series equations. In general, such a transformed set of equation (4.5) can be readily converted to a second kind matrix system of equations; in this example, it turns out that the exact solution is explicitly obtained (effectively the relevant matrix is diagonal).

First, we apply Abel’s integral transform to equation (4.1). From (3.7) by setting α=-1/2,β=η=1/2,


$$\int_{-1}^{t}\frac{P_{n}^{\left(0,0\right)}\left(x\right)}{{\left(t-x\right)}^{1/2}}dx=\sqrt{\pi }{\left(1+t\right)}^{1/2}\frac{\Gamma \left(n+1\right)}{\Gamma \left(n+3/2\right)}P_{n}^{\left(-1/2,1/2\right)}\left(t\right).$$

Applying this transform to the series (4.1), and accepting that the interchange of summation and integration is justified, we arrive at the transformed equation


$$\sum_{n=0}^{\infty }\frac{\Gamma \left(n+1\right)}{\Gamma \left(n+1/2\right)}a_{n}P_{n}^{\left(-1/2,1/2\right)}\left(x\right)=0,\;\;\;x\in \left(-1,x_{0}\right).$$

Turning to the second equation (4.2) of the dual series pair, the Abel integral representation for the Jacobi polynomial $P_{n}^{\left(0,0\right)}\left(x\right)$ is obtained from (3.6) with parameters α=β=0,η=1/2; it is essentially the inverse transform of the polynomial $P_{n}^{\left(-1/2,1/2\right)}\left(t\right)$ of the newly introduced basis:


$$P_{n}^{\left(0,0\right)}\left(x\right)=\frac{\Gamma \left(n+1\right)}{\sqrt{\pi }\Gamma \left(n+1/2\right)}\int_{x}^{1}\frac{{\left(1-t\right)}^{-1/2}P_{n}^{\left(-1/2,1/2\right)}\left(t\right)}{{\left(t-x\right)}^{1/2}}dt.$$

We substitute in equation (4.2), change the order of summation and integration and arrive at


$$\int_{x}^{1}\frac{{\left(1-t\right)}^{-1/2}}{{\left(t-x\right)}^{1/2}}\sum_{n=0}^{\infty }\frac{\Gamma \left(n+1\right)}{\Gamma \left(n+1/2\right)}a_{n}P_{n}^{\left(-1/2,1/2\right)}\left(t\right)dt=\sqrt{\pi }.$$

Denoting by F(t) the quantity


$$\sum_{n=0}^{\infty }\frac{\Gamma \left(n+1\right)}{\Gamma \left(n+1/2\right)}a_{n}P_{n}^{\left(-1/2,1/2\right)}\left(t\right),$$

we may recognize that the Abel integral equation


$$\int_{x}^{1}\frac{{\left(1-t\right)}^{-1/2}F(t)}{{\left(t-x\right)}^{1/2}}dt=\sqrt{\pi }$$

is invertible with the help of formula (2.5) to obtain


$${\left(1-t\right)}^{-1/2}F\left(t\right)=\frac{1}{\sqrt{\pi }}{\left(1-t\right)}^{-1/2}.$$

Combining these results, we obtain an equation of the desired form (4.5):


(4.6) $$F\left(t\right)=\sum_{n=0}^{\infty }\frac{\Gamma \left(n+1\right)}{\Gamma \left(n+1/2\right)}a_{n}P_{n}^{\left(-1/2,1/2\right)}\left(x\right)=\left\{ \begin{array}{l} 0,\;\;\;x\in \left(-1,x_{0}\right)\\ \frac{1}{\sqrt{\pi }},\;\;\;x\in \left(x_{0},1\right). \end{array}\right.$$

In order to complete the solution and determine the coefficients $a_{n}$, the piecewise constant function defined by the right-hand side of (4.6) may be expanded in terms of the complete orthonormal set of functions $P_{n}^{\left(-1/2,1/2\right)}\left(x\right)\left(n=0,1,2,\ldots \right)$. This is most conveniently done by returning to the trigonometric argument ($x=\cos\theta ,x_{0}=\cos\theta_{0}$), using relationship (3.2):


(4.7) $$\sum_{n=0}^{\infty }a_{n}\cos\left(n+\frac{1}{2}\right)\theta =\left\{ \begin{array}{l} \cos\frac{\theta }{2},\;\;\;\;\;\;\;\;\;\theta \in \left(0,\theta_{0}\right),\\ 0,\;\;\;\;\;\;\;\;\;\;\;\;\;\;\;\theta \in \left(\theta_{0,}\pi \right). \end{array}\right.$$

Recall that the orthogonal set B of functions ${\left\{\cos\left(n+\frac{1}{2}\right)\theta \right\}}_{n=0}^{\infty }$ is a complete basis for $L^{2}\left(0,\pi \right)$ and


$$\int_{0}^{\pi }\cos\left(n+\frac{1}{2}\right)\theta \cos\left(m+\frac{1}{2}\right)\theta d\theta =\frac{\pi }{2}\delta_{nm},$$

where $\delta_{nm}$ denotes the Kronecker delta. Thus, multiplying both parts of (4.7) by $\cos\left(m+\frac{1}{2}\right)\theta$ and integrating over $\left(0,\pi \right)$ we obtain the explicit analytic solution


(4.8) $$a_{n}=\frac{1}{\pi }\left\{\frac{\sin n\theta_{0}}{n}+\frac{\sin\left(n+1\right)\theta_{0}}{n+1}\right\},\;n>0;\;\;\;a_{0}=\frac{1}{\pi }\left(\theta_{0}+\sin\theta_{0}\right).$$

(This is equivalent to expanding the piecewise constant function defined by the right-hand side of (4.7) in the Fourier series with basis B and equating coefficients.) The potential field at any point in the interior and exterior of the shell is now easily computed from these coefficients (see [4]).

Of course, this simple example does not exhaust all the problems amenable to solution by the Abel integral transform method. It treats dual or triple series equations involving Jacobi polynomials $P_{n}^{\left(\alpha ,\beta \right)}\left(x\right)$ with arbitrary indexes α and β. However, some problems give rise to parameters α and β that prevent the direct use of the Abel transform method. This circumstance is overcome by employing Rodrigues’ formula (3.10). A detailed description of the mathematically justified solutions to various dual (triple) series and integral equations, involving the Jacobi polynomials, the associated Legendre polynomials $P_{n}^{m}\left(\cos\theta \right)$ with trigonometric argument, the trigonometric functions, or the Bessel functions, is given in [4, see ch. 2]. Previously, such equations were studied mostly in a formal fashion and by diverse methods; the Abel integral transform approach provides a common or universal tool for tackling such apparently disparate problems.

## 5. Electromagnetic wave scattering problems for slotted cylinders of arbitrary profile

Let us recall two well-known facts concerning wave scattering by two-dimensional objects, or more exactly, cylinders (open or closed) with axes aligned with the *z*-axis, of constant cross section and illuminated by a *z*-independent field. The Dirichlet BVP for the Helmholtz equation, posed on the two-dimensional cross section, simultaneously describes acoustic scattering by a soft obstacle and EM scattering of E-polarized waves by PEC obstacles; the Neumann BVP simultaneously describes acoustic scattering by a rigid obstacle and EM scattering of H-polarized waves by PEC obstacles. This interdisciplinary link means that solutions of an EM wave scattering problem may be derived from solutions of the corresponding acoustic problem by replacing the velocity potential U (or acoustic pressure p) with values proportional to axial components of EM field $E_{z}$ (Dirichlet problem) or $H_{z}$ (Neumann problem).

In this section, we discuss the solution of EM wave scattering problems for infinitely thin slotted PEC cylinders of arbitrary shape, bounded by smooth non-intersecting contours. The first key steps were developed as far back as 1985 in [31] and 1987 in [32]. Later, the comprehensive investigation of [33] mathematically proved all steps in the deduction of the solution, providing a rigorous foundation of the MAR in tackling mixed BVPs for the Helmholtz equation in two-dimensional space. Unfortunately, this Russian edition is very difficult to access; however, later publications (referenced below) partially expound the approach of [33].

As previously mentioned, the method of the Riemann–Hilbert problem, and the Abel integral transform method, applied to the mixed BVP problems for the Helmholtz equation in two-dimensional space, produce identical solutions. EM wave scattering problems for slotted cylinders of canonical circular and elliptic shapes have been rigorously solved by the Riemann–Hilbert approach. Diffraction of a plane electromagnetic wave by a circular cylinder with a longitudinal slot was first rigorously treated in [9] and extended to diffraction by a finite number of slotted cylinders in several publications (e.g. [34,35]); furthermore, the solution [11] was used to investigate the low-frequency resonance phenomenon in slotted cylinders [10]. The Riemann–Hilbert approach was also used to treat diffraction by slotted elliptic cylinders [36].

However, for a long time, the Abel integral transform method was not extensively used for practical problems of radio-engineering and applied acoustics. That can be partly attributed to the complexity of the solution, especially in the case of H-polarization. With the passage of time though, its merits have become increasingly apparent and attractive. They include solution stability in a wide frequency band (from low frequencies to quasi-optics); guaranteed convergence of the solution to the finite system of linear algebraic equations, obtained by truncation of the second kind matrix system obtained as the output of the method, to its exact solution as the truncation order $N_{tr}$ is increased indefinitely; the convergence is fast and the resulting solutions accurate. The assembly of the matrix elements is fast because all or nearly all elements are computed by the Fast Fourier Transform or recursive procedures. (The only exception occurs in some cases of H-polarization where calculations employing numerical differentiation and requiring controlled accuracy arise; however, this does not materially influence the total matrix fill time which remains a fast and accurate procedure.) This is in strong contrast to the Method of Moments (MoM) approach in solving integral equation formulations of scattering problems, where the assembly of the underlying matrix is resource intensive, due to the numerical integration procedures employed, and often severely limits the size of scattering problems that can be solved.

When the bounding contours of the open scatterer are of canonical circular or elliptic shape, a regularized solution to the wave scattering problem can be constructed employing eigenfunctions of the Helmholtz operator (see [4]). On the other hand, the apparatus of the single-layer or double-layer potentials is a natural starting point for contours of arbitrary shape. The deduction of the final form of the second kind infinite matrix system to be solved for EM plane wave scattering by a PEC arbitrary slotted cylinder, for both polarizations, is rather lengthy. This is due to the combination of several steps, of which the central step is inversion of two sets of dual series equations on the interval $\left[0,\pi \right]$—one involving the trigonometrical basis ${\left\{\cos n\theta \right\}}_{n=0}^{\infty }$ and the other the basis ${\left\{\sin n\theta \right\}}_{n=1}^{\infty }$. Although this step produces rather bulky expressions for the matrix elements, the advantages described above significantly outweigh this single drawback, particularly for cavity backed apertures for which purely numerical methods applied to the (untransformed) IEs produce matrix systems that are increasingly ill-conditioned as the underlying grid is refined (and there is no convergence to the exact solution).

**Figure 1.**
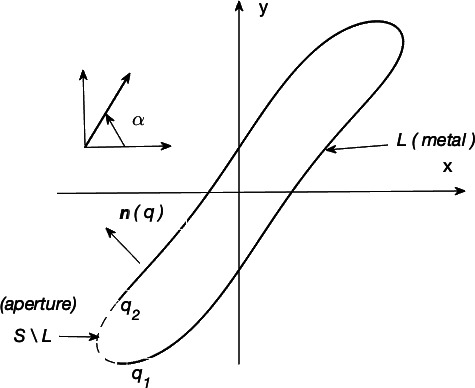
Scattering of an EM plane wave by a two-dimensional slotted PEC cavity of arbitrary cross section.

Consider the scatterer geometry shown in figure 1, a PEC cylindrical cavity of constant cross section with infinitely thin walls and a longitudinal slit, with axis aligned with the *z*-axis. The cavity cross section is bounded by an arbitrary open contour L, which is part of a smooth closed contour S. The aperture S\L is formed by the removal of the contour L from the closed contour S. The edges of the aperture are the points q_1_, q_2_. The formulation of the scattering problem is as follows. The structure is illuminated by a plane wave at an incident angle α. If the incident field is E-polarized (resp. H-polarized), only the *z*-component of the electric (resp. magnetic) field (incident, total or scattered) is non-zero, while the *z*-component of the magnetic (resp. electric) field (incident, total or scattered) is zero, and the other two magnetic (resp. electric) field components are derived from the single electric (resp. magnetic) field component. It is required to find the electric (resp. magnetic) scattered field component $E_{z}^{sc}$ (resp. $H_{z}^{sc}$), which along with electric (resp. magnetic) incident field $E_{z}^{0}$ (resp. $H_{z}^{0}$), forms the total electric (resp. magnetic) field $E_{z}^{tot}=E_{z}^{0}+E_{z}^{sc}$ (resp. $H_{z}^{tot}=H_{z}^{0}+H_{z}^{sc}$).

The following conditions, which are necessary and sufficient for solution uniqueness, should be satisfied: (i) the scattered field $E_{z}^{sc}\left(q\right)$$\left(or\;H_{z}^{sc}\left(q\right)\right)$ must satisfy the Helmholtz equation at each point $q\in R^{2}$, (ii) mixed boundary conditions should be satisfied on S, namely that $E_{z}^{tot}$ (resp. $H_{z}^{tot}$) be continuous at each point q on the aperture S\L and obey the Dirichlet (resp. Neumann) boundary condition at each point q on the PEC contour L, (iii) the scattered component obeys the Sommerfeld radiation condition $E_{z}^{sc}(q)=o\left({\left|q\right|}^{-1/2}\right)$ (resp. $H_{z}^{sc}(q)=o\left({\left|q\right|}^{-1/2}\right)$ as $\left|q\right|\to \infty$ ensuring that it behaves as an outgoing cylindrical wave at infinity, and (iv) the energy of the total EM field vector $\left(\vec{E},\vec{H}\right)$ should be bounded in any arbitrary cross-sectional area A including the edges,


(5.1) $$\iint_{A}^{,}\left\{\varepsilon_{0}{\left|\vec{E}\left(q\right)\right|}^{2}+\mu_{0}{\left|\vec{H}\left(q\right)\right|}^{2}\right\}dS_{q}<\infty ,$$

where $\varepsilon_{0}$ and $\mu_{0}$ denote the permittivity and permeability of free space, respectively; this condition automatically determines the singularity order of the surface current (or charges) densities at the sharp edges, usually known as the edge condition for these densities. Let us focus on the E-polarization and the H-polarization cases separately in the next two subsections.

### (a) Electromagnetic wave scattering problems for slotted cylinders: E- polarization

In the case of E-polarization, the scattered field is representable [37] as a single-layer potential of an unknown line current density $J_{z}$ to be found:


(5.2) $$E_{z}^{sc}(q)=ikZ_{0}\int_{L}G_{2}(q,p)J_{z}(p)\;dl_{p},\;\;\;q\in R^{2},$$

where k denotes the wavenumber, $Z_{0}$ the impedance $\sqrt{\mu_{0}/\varepsilon_{0}}$ of free space, $G_{2}\left(q,p\right)=-\frac{i}{4}H_{0}^{\left(1\right)}\left(k\left|p-q\right|\right)$ the free space Green’s function, and $dl_{p}$ the differential of arc length. Using the normalized density $Z\left(p\right)=ikZ_{0}J_{z}\left(p\right)$ defined for all p∈L and enforcing the Dirichlet boundary condition for the total field on the PEC surface, one arrives at the Fredholm IE of first kind


(5.3) $$\int_{L}G_{2}(q,p)Z(p)\;dl_{p}=-E_{z}^{0}(q),\;\;\;\;\;\;q\in L.$$

The complexity of equation (5.3) is governed by the nature of the singularity in its kernel which exhibits a weak singularity of logarithmic type. We remark that the corresponding kernel arising in the H-polarization case is hypersingular and introduces considerably more complexity in the analysis.

We assume that the contour S has a smooth parameterization given by a function $\eta \left(\theta \right)=\left\{x\left(\theta \right),y\left(\theta \right)\right\}(\theta \in \left[-\pi ,\pi \right])$, so that after its 2π periodic continuation, it is smooth on $\left(-\infty ,\infty \right)$ and η(-π)=η(π). The screen L is parametrized by subinterval $\left[-\theta_{0},\theta_{0}\right]$ while the slot $L^{\prime }$ is created by the removal of the segment corresponding to the subintervals $\left[-\pi ,-\theta_{0}\right]\cup \left[\theta_{0},\pi \right]$ (thus $S=L\cup \;L^{\prime }$). Given L, the choice of $L^{\prime }$ is not unique and may be chosen as convenient for the problem at hand. In this article we will assume, for simplicity, that the function $\eta \left(\theta \right)$ is infinitely differentiable; however, the validity of the analytical regularization procedure is usually ensured by requiring that $\eta \left(\theta \right)$ is twice (resp. thrice) continuously differentiable for E-polarization (resp. H-polarization). The differential of arc-length at p(τ) is


(5.4) $$dl_{p\left(\tau \right)}=l\left(\tau \right)d\tau =\sqrt{{\left(x^{\prime }\left(\tau \right)\right)}^{2}+{\left(y^{\prime }\left(\tau \right)\right)}^{2}}d\tau$$

and the parameterized form of (5.3) becomes


(5.5) $$\int_{-\theta_{0}}^{\theta_{0}}G_{2}\left(p\left(\theta \right),p(\tau )\right)Z\left(p(\tau )\right)l\left(\tau \right)d\tau =-E_{z}^{0}\left(p(\theta )\right)$$

for $\theta \in \left[-\theta_{0},\theta_{0}\right]$. Defining the (discontinuous) function z(τ) by


$$z\left(\tau \right)=\left\{ \begin{array}{l} Z\left(p\left(\tau \right)\right),\;\;\;\;\;\;\tau \in \left[-\theta_{0},\theta_{0}\right]\\ 0\;\;\;\;\;\;\;\;\;\;,\;\;\;\;\;\;\;\;otherwise, \end{array}\right.$$

we may replace (5.5) by


(5.6) $$\int_{-\pi }^{\pi }G_{2}\left(p\left(\theta \right),p\left(\tau \right)\right)z\left(\tau \right)l\left(\tau \right)d\tau =-E_{z}^{0}\left(p\left(\theta \right)\right)$$

for $\theta \in \left[-\theta_{0},\theta_{0}\right]$; outside of this interval (i.e. in the aperture), the requirement $z\left(\theta \right)=0$ is enforced.

The main steps in the analysis are as follows. First, split the integral kernel into singular and smooth parts; second, represent all kernels and functions by exponential Fourier series and deal with the kernel singularity analytically; third, derive the pair of joint dual series equations (one with cosine functions and the other with sine functions) for the Fourier coefficients of the unknown function *z*(θ); and finally, use the Abel integral transform to obtain a coupled set of infinite systems of linear algebraic equations of the second kind. The process is completed by numerical solution of the regularized system.

In the present E-polarization case, the properties of the Hankel function and the smoothness of the contour L enable the kernel to be split in the form


(5.7) $$G_{2}\left(p\left(\theta \right),p(\tau )\right)=-\frac{i}{4}H_{0}^{\left(1\right)}\left(k\left|p\left(\theta \right)-p\left(\tau \right)\right|\right)=\frac{1}{2\pi }ln\left|2\sin\frac{\theta -\tau }{2}\right|+H\left(\theta ,\tau \right),$$

where the remainder H is smooth and continuously differentiable with respect to θ and τ; thus, it may be expanded in a double Fourier series


(5.8) $$H\left(\theta ,\tau \right)=\frac{1}{2\pi }\sum_{m=-\infty }^{\infty }\sum_{n=-\infty }^{\infty }h_{nm}e^{i\left(n\theta -m\tau \right)},$$

where


(5.9) $$\sum_{m=-\infty }^{\infty }\sum_{n=-\infty }^{\infty }\left(1+m^{2}\right)\left(1+n^{2}\right){\left|h_{nm}\right|}^{2}<\infty .$$

Expansion of the relevant kernel, the unknown density and the incident field in their Fourier series leads to dual series equations. Introduce the expansions


$$z\left(\tau \right)=\sum_{n=-\infty }^{\infty }\xi_{n}e^{in\tau },E_{z}^{0}\left(q\left(\theta \right)\right)=\frac{1}{2\pi }\sum_{n=-\infty }^{\infty }f_{n}e^{in\theta },$$

and use the well-known expansion


$$\ln\left|2\sin\frac{\theta -\tau }{2}\right|=-\frac{1}{2}{\sum_{\begin{array}{c} n=-\infty \end{array}}^{\infty }}^{\prime }\frac{1}{|n|}e^{in(\theta -\tau )},$$

where the prime denotes omission of the term corresponding to n=0, to obtain


(5.10) $$\sum_{n=-\infty }^{\infty }\xi_{n}e^{in\theta }=0,\;\;\;\;\;\;\;\;\;-\pi \leq \theta <-\theta_{0}\;or\;\theta_{0}<\theta \leq \pi ,$$

and when $-\theta_{0}\leq \theta \leq \theta_{0}\;,$


(5.11) $${\sum_{n=-\infty }^{\infty }}^{\prime }\frac{1}{\left|n\right|}\xi_{n}e^{in\theta }-2\sum_{n=-\infty }^{\infty }\left(\sum_{p=-\infty }^{\infty }h_{np}\xi_{p}\right)e^{in\theta }=\sum_{n=-\infty }^{\infty }f_{n}e^{in\theta }.$$

The regularization of the dual series equations in (5.10), (5.11) and their transformations into well-conditioned coupled infinite systems of linear algebraic equations is well documented in [6]. They take the form


(5.12) $$\left\{ \begin{array}{ll} \left(I+H_{11}\right)\hat{x}+H_{12}\hat{y} & =b_{1}\\ H_{21}\hat{x}+\left(I+H_{22}\right)\hat{y} & =b_{2}, \end{array}\right.$$

where $\left\{\;\hat{x},\hat{y}\right\}\in l_{2}$ are infinite sequences of coefficients to be found, I is the identity operator, $H_{ij}\left(i,j=1,2\right)$ are completely continuous (compact) matrix operators in $l_{2}$, and $\left\{\;b_{1},b_{2}\right\}$ are known and lie in $l_{2}$.

The rate of decay of the coefficient matrices $h_{np}$ (see (5.9)) is sufficient to ensure that the transform of the initial dual series into the final matrix form (5.12) produces a Fredholm matrix system of second kind. Properties of such type of equations are well-studied (e.g. [38]); they are effectively solvable by a truncation method. The solution ${\left\{{\hat{x}}_{n}^{N_{tr}}\right\}}_{n=0}^{N_{tr}}{,\left\{{\hat{y}}_{n}^{N_{tr}}\right\}}_{n=1}^{N_{tr}}$ of the system (5.12), when truncated to one of finite order $2N_{tr}+1$, converges to the exact solution of the (untruncated) system (5.12), as $N_{tr}\to \infty$. A measure of the convergence rate is provided by the normalized truncation error


(5.13) $$e\left(N_{tr}\right)=\frac{\underset{n\leq N_{\mathrm{tr}}}{\max}\left\{\left|{\hat{x}}_{n}^{N_{tr}+1}-{\hat{x}}_{n}^{N_{tr}}\right|,\left|{\hat{y}}_{n}^{N_{tr}+1}-y_{n}^{N_{tr}}\right|\right\}}{\underset{n\leq N_{\mathrm{tr}}}{\max}\{\left|{\hat{x}}_{n}^{N_{tr}+1}\right|,\left|{\hat{y}}_{n}^{N_{tr}+1}\right|\}}$$

comparing the relative difference between solutions of successive truncations.

A detailed description of the method, the construction of the code and its validation against other numerical approaches (such as MoM) appeared in [39]. A variety of scatterers was examined. In §5c will be given an example illustrating the rapid convergence to the solution.

### (b) Electromagnetic wave scattering problems for slotted cylinders: H-polarization

Let us turn to the H-polarization case. The scattered field is sought as a double-layer potential


(5.14) $$H_{z}^{sc}\left(q\right)=\int_{L}^{,}\frac{\partial G_{2}\left(q,p\right)}{\partial n_{p}}Z\left(p\right)dl_{p},q\in R^{2}\backslash L,$$

where the function $Z\left(p\right)$ to be found is the jump of scattered magnetic field component across the contour L, i.e.


$$Z\left(p\right)=H_{z}^{sc}\left(p-0\right)-H_{z}^{sc}\left(p+0\right),\;\;\;\;p\in L.$$

It is proved in [21] that, under the conditions stated above which ensure uniqueness, the IE


(5.15) $$\underset{h\to \pm 0}{\lim}\frac{\partial }{\partial n_{q}}\int_{L}^{,}Z\left(p\right)\frac{\partial G_{2}\left(q+hn_{q},p\right)}{\partial n_{p}}dl_{p}=-\frac{\partial H_{z}^{0}\left(q\right)}{\partial n_{q}},q\in L$$

for the unknown function $Z\left(p\right)$ is obtained.

As mentioned in the previous subsection, the nature of the singularity in the kernel of (5.15) introduces considerably more complexity in its analysis than for the corresponding kernel arising in the E-polarization case. The kernel is hypersingular, so it is be expected that the solution for equation (5.15) is very much more difficult than that for (5.3); indeed, hypersingular kernels present one of the most difficult objects of study in the theory of integral equations.

Upon introducing the parameterization and its associated differential of arc-length (5.6), equation (5.15) becomes


(5.16) $$\underset{h\to \pm 0}{\lim}\frac{\partial }{\partial n_{p(\theta )}}\int_{{-\theta }_{0}}^{\theta_{0}}\frac{\partial G_{2}\left(p\left(\theta \right)+hn_{p\left(\theta \right)},p\left(\tau \right)\right)}{\partial n_{p\left(\tau \right)}}Z\left(p\left(\tau \right)\right)l\left(\tau \right)d\tau =-\frac{\partial H_{z}^{0}\left(p(\theta )\right)}{\partial n_{p(\theta )}}$$

for $\theta \in \left[-\theta_{0},\theta_{0}\right].$

In a similar way to that of the preceding subsection, we define the (discontinuous) function z(τ) by


$$z\left(\tau \right)=\left\{ \begin{array}{l} Z\left(p\left(\tau \right)\right),\;\;\;\;\;\;\tau \in \left[-\theta_{0},\theta_{0}\right]\\ 0\;\;\;\;\;\;\;\;\;\;,\;\;\;\;\;\;\;\;otherwise, \end{array}\right.$$

and replace (5.16) by


(5.17) $$\lim_{h\to \pm 0}\frac{\partial }{\partial n_{p\left(\theta \right)}}\int_{-\pi }^{\pi }\frac{\partial G_{2}\left(p\left(\theta \right)+hn_{p\left(\theta \right)},\;\;p\left(\tau \right)\right)}{\partial n_{p\left(\tau \right)}}z\left(\tau \right)l\left(\tau \right)d\tau =-\frac{\partial H_{z}^{0}\left(p\left(\theta \right)\right)}{\partial }n_{p}\left(\theta \right),$$

for $\theta \in \left[-\theta_{0},\theta_{0}\right]$; outside of this interval (i.e. on the aperture surface), the requirement $z\left(\theta \right)=0$ is enforced.

The main steps in the analysis follow the same path as for E-polarization. First, split the integral kernel into singular and smooth parts; second, represent all kernels and functions by exponential Fourier series and deal with the kernel singularity analytically; third, derive the pair of joint dual series equations (one with cosine functions and the other with sine functions) for the Fourier coefficients of the unknown function *z*(θ); and finally, use the Abel integral transform to obtain a coupled set of infinite systems of linear algebraic equations of the second kind. The process is completed by numerical solution of the regularized system.

Considering equation (5.17), the kernel, in the limit as h→±0 , produces the hypersingular kernel


(5.18) $$D_{0}\left(\vartheta ,\tau \right)\equiv \frac{\partial^{2}G_{2}\left(p\left(\theta \right),p\left(\tau \right)\right)}{\partial n_{p\left(\theta \right)}\partial n_{p\left(\tau \right)}};$$

it contains a non-integrable algebraic singularity of the form ${\left(\theta -\tau \right)}^{-2}$, as well as a logarithmic singularity, as θ-τ→0. It was proved in [32] that it has the decomposition


(5.19) $$D_{0}\left(\theta ,\tau \right)=\frac{1}{2\pi l\left(\theta \right)l\left(\tau \right)}\left[K\left(\theta ,\tau \right)-\frac{1}{4}{\left(\sin\frac{\theta -\tau }{2}\right)}^{-2}\right],$$

where the function $K\left(\theta ,\tau \right)$ has only a logarithmic singularity. Recognizing that


$$\frac{\partial^{2}}{\partial \theta^{2}}\log\left|2\sin\frac{\theta -\tau }{2}\right|=-\frac{1}{4}{\left(\sin\frac{\theta -\tau }{2}\right)}^{-2},$$

we obtain the equivalent *integral-differential equation*


(5.20) $$\frac{d^{2}}{d\theta^{2}}\int_{-\pi }^{\pi }z\left(\tau \right)\log\left|2\sin\frac{\theta -\tau }{2}\right|d\tau +\int_{-\pi }^{\pi }z\left(\tau \right)K\left(\theta ,\tau \right)d\tau =F\left(\theta \right),\;\;\;\;\;\;\;\;\theta \in \left[-\theta_{0},\theta_{0}\right],$$

where the kernel $K\left(\theta ,\tau \right)$ and the function $F\left(\theta \right)$ are defined by


$$K\left(\theta ,\tau \right)=2\pi l\left(\theta \right)l\left(\tau \right)D_{0}\left(\theta ,\tau \right)+\frac{1}{4}{\left(\sin\frac{\theta -\tau }{2}\right)}^{-2},\;\;\;\;\;\;F\left(\theta \right)=-2\pi l\left(\theta \right)\frac{\partial H_{z}^{0}\left(p\left(\theta \right)\right)}{\partial n_{p\left(\theta \right)}}.$$

In turn, the kernel $K\left(\theta ,\tau \right)$ is decomposed into a purely singular part and a smooth part, in the form


(5.21) $$K\left(\theta ,\tau \right)=-\frac{1}{2}kl\left(\theta \right)\cdot kl\left(\tau \right)\log\left|2\sin\frac{\theta -\tau }{2}\right|+K_{s}\left(\vartheta ,\tau \right).$$

The function $K_{s}\left(\theta ,\tau \right)$is smooth in the sense that, after its 2π-periodic continuation with respect to both variables in the plane, both first partial derivatives are continuous, and its second partial derivatives have only logarithmic singularities. It may be expanded in a double Fourier series


(5.22) $$K_{s}\left(\theta ,\tau \right)=\sum_{m=-\infty }^{\infty }\sum_{n=-\infty }^{\infty }\kappa_{nm}^{s}e^{i(n\theta -m\tau )}$$

satisfying the same condition (5.9) as the corresponding series in the E-polarization case. Some care is needed in the evaluation of $K_{s}\left(\vartheta ,\tau \right)$ at ϑ=τ. Further details can be found in [32] as well as a schematic description in [40]. An improved decomposition of the kernel $K\left(\theta ,\tau \right)$ together with valuable advice on the organization of the numerical algorithms has been given to us by Yu A. Tuchkin (author of [32]) in private communication; an extended description is given in [41]. This version significantly facilitated the successful numerical implementation of the solution for the H-polarized wave scattering problem.

Expansion of the relevant kernel, the unknown density and the incident field in their Fourier series leads to dual series equations. Thus, expand the unknown density and the normal derivative of the incident field as


$$z(\tau )=\sum_{n=-\infty }^{\infty }\xi_{n}e^{in\tau },\;\;F\left(\theta \right)=\sum_{n=-\infty }^{\infty }f_{n}^{\prime }e^{in\theta },$$

insert the decompositions of $K\left(\theta ,\tau \right)$ and $K_{s}\left(\theta ,\tau \right)$ into (5.17) and expand in a double Fourier series to obtain a dual series of the form


(5.23) $$\sum_{n=-\infty }^{\infty }\left\{\left|n\right|\xi_{n}+2\sum_{m=-\infty }^{\infty }\kappa_{nm}\xi_{m}\right\}e^{in\theta }=\sum_{n=-\infty }^{\infty }f_{n}^{\prime }e^{in\theta },\;\;\;\;\;\;\theta \in \left[-\theta_{0},\theta_{0}\right],$$

(5.24) $$\sum_{n=-\infty }^{\infty }\xi_{n}e^{in\theta }=0,\;\;\;\;\;\;\;\;\theta \in \left[-\pi ,-\theta_{0}\right]\cup \left[\theta_{0},\pi \right].$$

The regularization of the dual series equations in (5.23), (5.24) and their transformations into well-conditioned coupled infinite systems of linear algebraic equations is well documented in [4]. The system takes the same form (5.12) as achieved in the E-polarization case, namely


$$\left\{ \begin{array}{ll} \left(I+H_{11}\right)\hat{x}+H_{12}\hat{y} & =b_{1}\\ H_{21}\hat{x}+\left(I+H_{22}\right)\hat{y} & =b_{2}, \end{array}\right.$$

where $\left\{\;\hat{x},\hat{y}\right\}\in l_{2}$ are infinite sequences of coefficients to be found, I is the identity operator, $H_{ij}\left(i,j=1,2\right)$ are completely continuous (compact) matrix operators in $l_{2}$, and $\left\{\;b_{1},b_{2}\right\}$ are known and lie in $l_{2}$. (The matrix operators $H_{ij}$ are of course different to those obtained in the E-polarization case.)

Let us illustrate how the coupled system arises. The preliminary step converts the dual series (5.23), (5.24) to trigonometric form on the intervals $\left[0,\theta_{0}\right]$ and $\left[\theta_{0},\pi \right]$. Using the symmetry of the interval, (5.24) is equivalent to


$$\sum_{n=-\infty }^{\infty }\xi_{n}\left(e^{in\theta }\pm e^{-in\theta }\right)=0,\;\;\;\;\;\;\;\;\;\theta_{0}<\theta \leq \pi ,$$

so setting $x_{n}=\xi_{n}+\xi_{-n}$, $y_{n}=\xi_{n}-\xi_{-n}$, (5.24) is equivalent to the pair of equations


(5.25) $$\sum_{n=1}^{\infty }x_{n}\cos n\theta =-\xi_{0},\;\;\sum_{n=1}^{\infty }y_{n}\sin n\theta =0,\;\;\theta_{0}<\theta \leq \pi .$$

In a similar way, (5.23) is equivalent to the pair


(5.26) $$\sum_{n=1}^{\infty }nx_{n}\cos n\vartheta +\sum_{n=1}^{\infty }\left\{\sum_{m=1}^{\infty }K_{nm}^{\prime }x_{m}+K_{nm}^{{\;}^{\prime \prime }}y_{m}\right\}\cos n\theta =K_{1}\left(\theta \right)\xi_{0}+f_{0}^{\prime }+\sum_{n=1}^{\infty }\left(f_{n}^{\prime }+f_{-n}^{\prime }\right)\cos n\theta$$

$$\sum_{n=1}^{\infty }ny_{n}\sin n\vartheta +\sum_{n=1}^{\infty }\left\{\sum_{m=1}^{\infty }K_{nm}^{{\;}^{\prime \prime \prime }}y_{m}+K_{nm}^{{\;}^{⁗}}x_{m}\right\}\sin n\theta =K_{2}\left(\theta \right)\xi_{0}+\sum_{n=1}^{\infty }\left(f_{n}^{\prime }-f_{-n}^{\prime }\right)\sin n\theta ,$$

where $\theta \in \left(0,\theta_{0}\right)$; here $K_{nm}^{\prime },\ldots ,K_{nm}^{\prime \prime \prime \prime }$ are simple linear combinations of the coefficients $\kappa_{nm}$, while $K_{1}\left(\theta \right),K_{2}\left(\theta \right)$ have Fourier expansions simply expressible in terms of the coefficients $\kappa_{nm}$. The Jacobi polynomial representations (3.1), (3.3) may now be employed. The two sets of dual series (5.25) and (5.26) are not independent—the unknowns $x_{n},y_{n}$ occur in both sets and so are coupled; however the rate of convergence of the coefficients $K_{nm}^{\prime },\ldots ,K_{nm}^{{\;}^{\prime \prime \prime }}$ is sufficient to allow us to treat the terms containing these coefficients as a perturbation to the dual series in which these terms are omitted.

The general solution for dual series of this trigonometric form is discussed in [4]. Let us illustrate the treatment of the sinusoidal case by considering a slightly more general, but notationally simpler, form, chosen to make the perturbative structure clearer. We consider the system of dual series equations


(5.27) $$\sum_{n=1}^{\infty }\left(y_{n}\left(1-q_{n}\right)-c_{n}\right)\sin n\theta =0,\;\;\theta_{0}<\theta \leq \pi ,$$

(5.28) $$\sum_{n=1}^{\infty }n\left(y_{n}\left(1-r_{n}\right)-h_{n}\right)\sin n\theta =0,\theta \in \left(0,\theta_{0}\right),$$

where appropriate choices of $c_{n},h_{n}$ and $q_{n},r_{n}$ are made in order to represent the various Fourier expansions and the coupling terms; it is required that $q_{n},r_{n}\to 0$ as n→∞. (In fact, $q_{n}=c_{n}=0$.) In this form the sequence of Abel transforms is made more transparent. Insert the representation (3.3), set $z=\cos\theta ,z_{0}=\cos\theta_{0}$, and obtain


(5.29) $$\sum_{n=1}^{\infty }\left(y_{n}\left(1-q_{n}\right)-c_{n}\right)\frac{\Gamma \left(n+1\right)}{\Gamma \left(n+\frac{1}{2}\right)}P_{n-1}^{\left(\frac{1}{2},\frac{1}{2}\right)}\left(z\right)=0,\;\;\;\;\;\;\;\;-1<z\leq z_{0},$$

(5.30) $$\sum_{n=1}^{\infty }n\left(y_{n}\left(1-r_{n}\right)-h_{n}\right)\frac{\Gamma \left(n+1\right)}{\Gamma \left(n+\frac{1}{2}\right)}P_{n-1}^{\left(\frac{1}{2},\frac{1}{2}\right)}\left(z\right)=0,\;\;\;\;\;\;\;\;z_{0}<z\leq 1.$$

First, use (3.8) to integrate (5.29) with weight ${\left(1-z\right)}^{\frac{1}{2}}$ on (z,1), obtaining


(5.31) $${\left(1-z\right)}^{\frac{3}{2}}\;\sum_{n=1}^{\infty }\frac{n}{n+\frac{1}{2}}\left(y_{n}\left(1-r_{n}\right)-h_{n}\right)\frac{\Gamma \left(n+1\right)}{\Gamma \left(n+\frac{1}{2}\right)}P_{n-1}^{\left(\frac{3}{2},-\frac{1}{2}\right)}\left(z\right)=0,\;\;\;\;\;\;z_{0}<z\leq 1.$$

Next insert, using (3.6),


(5.32) $$P_{n-1}^{\left(\frac{3}{2},-\frac{1}{2}\right)}(z)=\frac{{\left(1-z\right)}^{-\frac{3}{2}}\Gamma \left(n+\frac{3}{2}\right)}{\Gamma \left(\frac{1}{2}\right)\Gamma \left(n+1\right)}\int_{z}^{1}\frac{\left(1-x\right)P_{n-1}^{\left(1,0\right)}\left(x\right)}{{\left(x-z\right)}^{\frac{1}{2}}}dx$$

to obtain the homogeneous form of Abel’s IE

(5.33) $$\int_{z}^{1}\frac{\left(1-x\right)U\left(x\right)}{{\left(x-z\right)}^{\frac{1}{2}}}dx=0,\;\;where\;\;U\left(x\right)=\sum_{n=1}^{\infty }n\left(y_{n}\left(1-r_{n}\right)-h_{n}\right)P_{n-1}^{\left(1,0\right)}\left(x\right).$$

(As previously mentioned, the interchange of integration and summation in these steps is rigorously justified.) The unique solution of this homogeneous IE is (1−x)U(x)≡0, and we deduce


(5.34) $$\sum_{n=1}^{\infty }n\left(y_{n}\left(1-r_{n}\right)-h_{n}\right)P_{n-1}^{\left(1,0\right)}\left(z\right)=0,\;\;\;\;\;\;\;\;\;\;\;z_{0}<z\leq 1.$$

On the other hand, inserting the representation


(5.35) $$P_{n-1}^{\left(\frac{3}{2},-\frac{1}{2}\right)}(z)=\frac{{\left(1+z\right)}^{-\frac{1}{2}}\Gamma \left(n+\frac{1}{2}\right)}{\Gamma \left(\frac{1}{2}\right)\Gamma \left(n\right)}\int_{-1}^{z}\frac{{\left(1+x\right)}^{0}P_{n-1}^{\left(1,0\right)}\left(x\right)}{{\left(z-x\right)}^{\frac{1}{2}}}dx$$

into (5.29) gives another homogeneous Abel IE


(5.36) $$\int_{-1}^{z}\frac{V\left(x\right)}{{\left(z-x\right)}^{\frac{1}{2}}}dx=0,\;\;where\;\;V\left(x\right)=\sum_{n=1}^{\infty }n\left(y_{n}\left(1-q_{n}\right)-c_{n}\right)P_{n-1}^{\left(1,0\right)}\left(x\right).$$

Invoking again the uniqueness of the solution of this IE, we obtain


(5.37) $$\sum_{n=1}^{\infty }n\left(y_{n}\left(1-q_{n}\right)-c_{n}\right)P_{n-1}^{\left(1,0\right)}\left(z\right)=0,\;\;\;\;\;\;\;\;\;\;\;\;-1<z\leq z_{0}.$$

Thus, the dual series have been converted to a dual series pair in the basis ${\left\{P_{n-1}^{\left(1,0\right)}\left(z\right)\right\}}_{n=1}^{\infty }$ ; the decay rate of terms is the same (namely O(n)). These equations are now readily converted to a matrix system of second kind. Setting $n\left\{y_{n}{,c}_{n},h_{n}\right\}$ = $\left\{\hat{y_{n}}\hat{{,c}_{n}},\hat{h_{n}}\right\},$ we have


(5.38) $$\sum_{k=1}^{\infty }\hat{y_{k}}P_{k-1}^{\left(1,0\right)}\left(z\right)=\left\{ \begin{array}{l} \;F_{1}\left(z\right),\;\;-1<z\leq z_{0}\\ F_{2}\left(z\right),\;\;\;\;z_{0}<z\leq 1, \end{array}\right.$$

where $F_{1}\left(z\right)=\sum_{m=1}^{\infty }{(q}_{m}\hat{y_{m}}+\hat{c_{m}})P_{m-1}^{\left(1,0\right)}\left(z\right)$ and $F_{2}\left(z\right)=\sum_{m=1}^{\infty }{(r}_{m}\hat{y_{m}}+\hat{h_{m}})P_{m-1}^{\left(1,0\right)}\left(z\right)$. Recalling that the weight function associated with the orthogonal set of Jacobi polynomials $P_{k}^{\left(\alpha ,\beta \right)}\left(z\right)$ is ${\left(1-z\right)}^{\alpha }{\left(1+z\right)}^{\beta }$ , we multiply by $(1-z)P_{s-1}^{\left(1,0\right)}\left(z\right)$ and integrate over the complete interval (-1,1) to obtain, for each s=1,2,3,…,


(5.39) $$h_{s-1}^{\left(1,0\right)}{\hat{y}}_{s}=\sum_{m=1}^{\infty }\left(q_{m}{\hat{y}}_{m}+{\hat{c}}_{m}\right)\left(\delta_{sm}h_{s-1}^{\left(1,0\right)}-Q_{s-1,m-1}^{\left(1,0\right)}(z_{0})\right)+\sum_{m=1}^{\infty }\left(r_{m}{\hat{y}}_{m}+{\hat{h}}_{m}\right)Q_{s-1,m-1}^{\left(1,0\right)}(z_{0}).$$

where $Q_{s-1,m-1}^{\left(1,0\right)}\left(z_{0}\right)$ denotes the so-called incomplete scalar product


$$\int_{z_{0}}^{1}(1-z)P_{s-1}^{\left(1,0\right)}\left(z\right)P_{m-1}^{\left(1,0\right)}\left(z\right)dz$$

and $h_{s-1}^{\left(1,0\right)}$ denotes the weighted square norm of the Jacobi polynomial,


$$\int_{-1}^{1}\left(1-z\right){\left(P_{s-1}^{\left(1,0\right)}\left(z\right)\right)}^{2}dz.$$

The infinite set (5.39) of equations with indices s=1,2,3,…, represents a system of linear equations. The properties of the incomplete scalar product are discussed in [4]. The recursive relationships for their evaluation make the assembly of the matrix coefficients very efficient and rapid.

A similar process applied to the cosine dual series results in a similarly transformed set of linear equations. Both systems are coupled because the coefficients $c_{n},h_{n}$ and $q_{n},r_{n}$ contain contributions from the variables $x_{n},y_{n}$. After a simple rescaling of the variables $\hat{x_{n}}\hat{{,y}_{n}}$, we arrive at the final form (5.12). The rate of decay of the coefficients $\kappa_{nm}$ ensures that the matrix operators $H_{ij}\left(i,j=1,2\right)$ are completely continuous (compact) operators in $l_{2}$, and the system (5.39) is a Fredholm matrix system of second kind. The publication [42] gives a short but instructive account of the process.

As mentioned in the preceding subsection, this second kind system of equation (5.39) is effectively solvable by a truncation method. The solution ${\left\{{\hat{x}}_{n}^{N_{tr}}\right\}}_{n=0}^{N_{tr}}{,\left\{{\hat{y}}_{n}^{N_{tr}}\right\}}_{n=1}^{N_{tr}}$ of the system (5.12) corresponding to the equation (5.39) in the H-polarization case when truncated to one of finite order $2N_{tr}+1$, converges to the exact solution of the (untruncated) system (5.12), as $N_{tr}\to \infty$. The same measure of the convergence rate is provided by the normalized truncation error (5.13), comparing the relative difference between solutions of successive truncations.

### (c) Electromagnetic wave scattering for slotted cylinders: illustrative results

**Figure 2.**
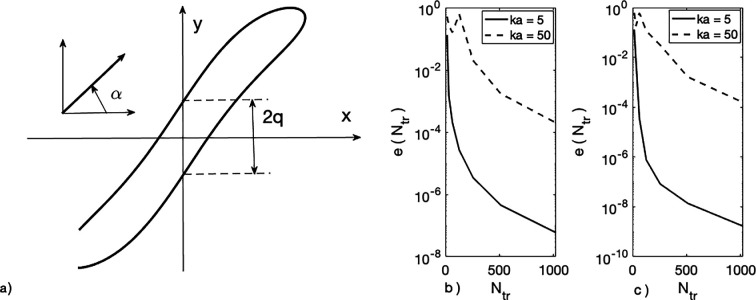
(*a*) The duct-like structure with q=0.3 and $\theta_{0}=150^{0}$; incidence angle $\alpha =0^{0}$. Truncation error: (*b*) TM-case; (*c*) TE-case.

In practice, the convergence rate of the solutions of the truncated systems to the exact solution of the infinite system (5.12) is fast. It may be illustrated by results obtained for the duct-like structure (see figure 2*a*) with parameterization dependent upon constants $a\left(=1\right),$q (0<q<1) and $\theta_{0}$, specifying the sharp edge:


(5.40) $$x(\theta )=a\cos\theta ,\;y(\theta )=a\left[\tan^{-1}\left(\frac{3}{2}\cos\theta \right)+q\sin\theta \right],-\theta_{0}<\theta <\theta_{0}.$$

**Figure 3.**
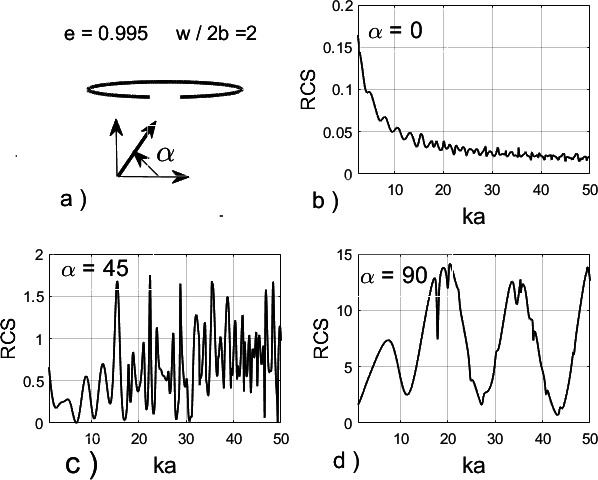
(*a*) The slotted elliptical cylinder. Normalized RCS, $\sigma_{B}$ as a function of relative wave number ka at various angles α of incidence: (*b*) $\alpha =0^{0}$ ; (*c*) $\alpha =45^{0}$; (*d*) $\alpha =90^{0}$.

The truncation errors are shown for both TM- and TE-incidence at three values of ka, namely 5, 20 and 50. They exhibit a similar and rather fast convergence rate. Computed physical characteristics of high accuracy are guaranteed. As an example, figure 3 shows the spectral dependence of the normalized radar cross section (RCS, $\sigma_{B}$) for an elliptical cylinder of high eccentricity e=0.995 (figure 3*a*), having minor major semi-axes b and a, and a slot of relative width $w_{2b}=w/2b=2$. The RCS for a TM-polarized plane wave incident at angles $\alpha =0^{0},45^{0}$ and $90^{0}$ is shown in figure 3*b*–*d*. The efficiency of our method enables a fine resolution of the RCS across the band 0<ka≤50, particularly where the response varies rapidly.

### (d) Electromagnetic wave scattering for slotted cylinders: applications and extensions

The regularized solutions obtained for both polarizations in §5a,b enabled the accurate solution of several practical problems of electrical engineering. For example, diffraction by a grating of finite extent was examined in [42]; and the impact of the addition of flanges to a rectangular cavity was quantified in [43]. The Abel transform approach was used extensively by Ionniadou [44] to analyse the scattering of a TE-polarized plane wave by a circular PEC slotted cylinder, enclosing two eccentric cylindrical dielectric layers. In [45], Ionniadou *et al.* extended the approach to scattering from the same PEC cylindrical structure, but enclosing an arbitrary number of eccentric dielectric cylinders, embedded in a dielectric sheath; both polarizations were considered.

The extension of the basic problem to multiple structures of cavity type (and arbitrary cross section) requires no additional mathematical tools. The scattering by a number *M* of slotted cylinders may be modelled by a system of *M* coupled IEs incorporating the interaction of each cylinder with the others as well as its own self-interaction. In each IE, only the self-interaction term has a singular kernel; the remaining $\left(M-1\right)$ terms may be viewed as smooth perturbations of the exciting field. The resulting regularized system is of second kind: see, for example [46]. An application to tuning a microwave cavity by a thin wire is described in [47].

Another interesting extension is the determination of the complex eigenvalues of a cavity structure, corresponding to the complex values *k* at which the homogeneous form of (5.3) or (5.15) has a non-trivial solution. This is equivalent to finding non-trivial solutions to the homogeneous form of the regularized (infinite) matrix system (5.12). Approximations to these complex eigenvalues of increasing accuracy are obtained by examining the roots of the determinantal equation, as the order of the truncated matrix is increased. An iterative method for obtaining these complex eigenvalues was successfully employed in [48–51]. In [52], the evolution of the complex eigenvalues of slotted metallic elliptic cavities, as the slot size increases, was examined in both polarizations.

## 6. Electromagnetic wave scattering by three-dimensional open shells

In this section, we turn to the Abel integral transform and its role for solving problems of EM wave scattering from three-dimensional open shells. Two preliminary remarks are in order. First, in contrast to the two-dimensional situation, such problems are not reducible to the corresponding acoustical problem, whether the shell is closed or open. The second note is less trivial. For closed axisymmetric shells illuminated by an EM field, comprising E- and H-polarized contributions, the scattered field of each polarization is independent of the other; when the axisymmetric shell is open, the scattered fields of different polarizations become coupled. A simple example is seen in the EM plane wave scattering from a PEC sphere with a circular aperture, at normal incidence (see [5,53,54]).

To date, the Abel integral transform has been successfully employed where the three-dimensional structure comprising an open shell or multiple open shells has rotational symmetry, i.e. is axisymmetric. Let us discuss the simplest structures—PEC spheres with one or two circular apertures or with an equatorial slit. The structure is excited by a normally incident wave emanating from axially positioned electric (or magnetic) vertical and horizontal dipoles. Other possible incident fields include a Huygens source comprising crossed electric and magnetic dipoles with moments of equal amplitude; in studying the spherical reflector antennas, the customary form of a Complex Huygens Source with beam-like radiation was employed.

**Figure 4.**
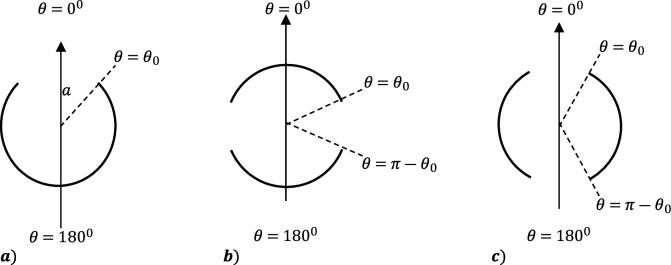
Cross sections of spherical shells with (*a*) a single circular aperture; (*b*) an equatorial slit; (*c*) two symmetric circular apertures.

Let us show schematically how the Abel integral transform method may be applied to derive the solution for plane wave scattering problem from a PEC sphere with a circular aperture (figure 4*a*). Since the surface of the shell of radius a and subtended aperture angle $\vartheta_{0}$ partially matches the coordinate surface ($r=a,\vartheta \in (\vartheta ,\pi ),\varphi \in \left(0,2\pi \right)$) in the spherical coordinate system r,ϑ,φ, it is natural to employ the method of separation of variables to formulate the solution of the Maxwell equations. The symmetry of the employed excitation sources imposes the same symmetry on the scattered field, so that the general solution (before the boundary conditions are enforced) contains only one non-zero azimuthal harmonic with index m=1: the general term of the series solution involves only the associated Legendre polynomials ${\left\{P_{n}^{1}\left(\cos\vartheta \right)\right\}}_{n=1}^{\infty }$.

The EM field may be described by two scalar functions, the electric $\left(U\left(r,\theta ,\varphi \right)\right)$ and magnetic ($V\left(r,\theta ,\varphi \right))$ Debye potentials. Fulfilment of the boundary conditions for the tangential electric field components $E_{\theta },E_{\varphi }$ at the metallic surface and continuity conditions for the tangential magnetic components $H_{\theta },H_{\varphi }$ on the aperture surface $\left(\vartheta \in \left({0,\vartheta }_{0}\right)\right)$ leads to a pair of coupled dual series equations involving the associated Legendre functions $P_{n}^{1}\left(\cos\vartheta \right)$. These equations contain two asymptotically small parameters


(6.1) $$\varepsilon_{n}=1+ika\frac{2n+1}{n\left(n+1\right)}\psi_{n}^{\prime }\left(ka\right)\varsigma_{n}^{\prime }\left(ka\right),\;\;\mu_{n}=1-\frac{i}{ka}\left(2n+1\right)\psi_{n}\left(ka\right)\varsigma_{n}\left(ka\right),$$

where


$$\psi_{n}\left(x\right)=\sqrt{\frac{\pi x}{2}}J_{n+1/2}\left(x\right),\varsigma_{n}\left(x\right)=\sqrt{\frac{\pi x}{2}}H_{n+1/2}^{\left(1\right)}\left(x\right)$$

are the spherical Bessel functions in Debye notation; as $n\to \infty ,\varepsilon_{n},\mu_{n}=O\left({\left(\frac{ka}{n}\right)}^{2}\right)$. After introducing the modified ‘electric’ ${\left\{x_{n}\right\}}_{n=1}^{\infty }$ and ‘magnetic’ ${\left\{y_{n}\right\}}_{n=1}^{\infty }$ Fourier coefficients, one arrives at the following coupled dual series equations (for details, see [5]):


(6.2) $$\sum_{n=1}^{\infty }x_{n}P_{n}^{1}\left(\cos\theta \right)=-ikaB_{1}\tan\frac{1}{2}\theta +\sum_{n=1}^{\infty }x_{n}{\varepsilon_{n}P}_{n}^{1}\left(\cos\theta \right),\theta \in \left(0,\theta_{0}\right)$$

(6.3) $$\sum_{n=1}^{\infty }\frac{2n+1}{n\left(n+1\right)}x_{n}P_{n}^{1}\left(\cos\theta \right)=B_{3}\cot\frac{1}{2}\theta +\sum_{n=1}^{\infty }i^{n}\frac{2n+1}{n\left(n+1\right)}\cdot \frac{P_{n}^{1}\left(\cos\theta \right)}{\varsigma_{n}^{`}\left(ka\right)},\theta \in \left(\theta_{0},\pi \right)$$

(6.4) $$\sum_{n=1}^{\infty }\frac{y_{n}}{n(n+1)}P_{n}^{1}(\cos\theta )=-\frac{i}{ka}B_{1}\tan\frac{1}{2}\theta +\sum_{n=1}^{\infty }\frac{y_{n}}{n(n+1)}\mu_{n}P_{n}^{1}(\cos\theta ),\;\;\theta \in \left(0,\theta_{0}\right)$$

(6.5) , $$\sum_{n=1}^{\infty }\frac{2n+1}{n(n+1)}y_{n}P_{n}^{1}(\cos\theta )=-iB_{3}\cot\frac{1}{2}\theta +\sum_{n=1}^{\infty }i^{n}\frac{\left(2n+1\right)}{n(n+1)}\cdot \frac{P_{n}^{1}(\cos\theta )}{\varsigma_{n}(ka)},\;\;\theta \in \left(\theta_{0},\pi \right).$$

In these equations, $B_{1}$ and $B_{3}$ denote polarization coupling constants, which appear when the boundary conditions are imposed.

It is well-known that the Legendre polynomials $P_{n}\left(\cos\vartheta \right)$ may be represented by the Mehler–Dirichlet integral [28]


(6.6) $$P_{n}\left(\cos\theta \right)=\frac{\sqrt{2}}{\pi }\int_{0}^{\theta }\frac{\cos\left(n+\frac{1}{2}\right)\phi }{\sqrt{\cos\phi -\cos\theta }}d\phi =\frac{\sqrt{2}}{\pi }\int_{\theta }^{\pi }\frac{\sin\left(n+\frac{1}{2}\right)\phi }{\sqrt{\cos\theta -\cos\phi }}d\phi .$$

This is simply Abel’s integral representation (see §3) of the Legendre polynomials $P_{n}\left(\cos\theta \right)$ in trigonometric form. The corresponding representation of the associated Legendre polynomials $P_{n}^{1}\left(\cos\theta \right)$ is readily deduced, using results of §1 (see also [4]):


(6.7) $$P_{n}^{1}\left(\cos\theta \right)=\frac{2\sqrt{2}}{\pi }\cdot \frac{1}{\sin\theta }\cdot \frac{n\left(n+1\right)}{2n+1}\left\{ \begin{array}{l} \int_{0}^{\theta }\frac{\sin\left(n+\frac{1}{2}\right)\phi \sin\phi }{\sqrt{\cos\phi -\cos\theta }}d\phi \\ -\int_{\theta }^{\pi }\frac{\cos\left(n+\frac{1}{2}\right)\phi \sin\phi }{\sqrt{\cos\theta -\cos\phi }}d\phi . \end{array}\right.$$

Also, the trigonometric functions $\tan\frac{1}{2}\theta$ and $\cot\frac{1}{2}\theta$ may be represented as Mehler–Dirichlet integrals:


(6.8) $$\tan\frac{1}{2}\theta =\frac{1-\cos\theta }{\sin\theta }=\frac{\left\{P_{0}\left(\cos\theta \right)-P_{1}\left(\cos\theta \right)\right\}}{\sin\theta }=\frac{2\sqrt{2}}{\pi }\cdot \frac{1}{\sin\theta }\int_{0}^{\theta }\frac{\sin\frac{\phi }{2}\sin\phi d\phi }{\sqrt{\cos\phi -\cos\theta }}$$

(6.9) $$\cot\frac{1}{2}\theta =\frac{1+\cos\theta }{\sin\theta }=\frac{\left\{P_{0}\left(\cos\theta \right)+P_{1}\left(\cos\theta \right)\right\}}{\sin\theta }=\frac{2\sqrt{2}}{\pi }\cdot \frac{1}{\sin\theta }\int_{0}^{\theta }\frac{\cos\frac{\phi }{2}\sin\phi d\phi }{\sqrt{\cos\phi -\cos\theta }}.$$

Let us briefly sketch the main steps in the regularization of the dual series equations. First integrate both parts of (6.2), keeping in mind that


$$\int_{0}^{\vartheta }P_{n}^{1}(\cos\theta )\;d\theta =-P_{n}(\cos\vartheta ),\;\;\int_{0}^{\vartheta }\tan\frac{\theta }{2}d\theta =-2\log\left(\cos\frac{\vartheta }{2}\right)$$

substitute the Mehler–Dirichlet integrals


$$P_{n}(\cos\vartheta )=\frac{\sqrt{2}}{\pi }\int_{0}^{\vartheta }\frac{\cos(n+\frac{1}{2})\theta }{\sqrt{\cos\theta -\cos\vartheta }}\;d\theta ,\;\;\log\left(\cos\frac{\vartheta }{2}\right)=-\frac{1}{\sqrt{2}\pi }\int_{0}^{\vartheta }\frac{\theta \sin\frac{\theta }{2}}{\sqrt{\cos\theta -\cos\vartheta }}d\theta ,$$

into the integrated equation and interchange the order of summation and integration to obtain a homogeneous Abel integral equation. The equation (6.3) is directly transformed into a homogeneous Abel integral equation by using the Abel integrals for $P_{n}^{1}\left(\cos\theta \right)$ and $\cot\frac{\theta }{2}$. As a result, we obtain a function (given by its Fourier expansion), which is piecewise continuously defined at the complete interval $\left(0,\pi \right)$, via


(6.10) $$\sum_{n=1}^{\infty }x_{n}\cos\left(n+\frac{1}{2}\right)\theta =\left\{ \begin{array}{l} ikaB_{1}\theta \sin\frac{\theta }{2}+C\cos\frac{\theta }{2}+\sum_{n=1}^{\infty }x_{n}\varepsilon_{n}\cos\left(n+\frac{1}{2}\right)\theta ,\;\;\;\theta \in \left(0,\theta_{0}\right)\\ B_{3}\cos\frac{\theta }{2}+\sum_{n=1}^{\infty }\frac{i^{n}}{\varsigma_{n}^{\prime }\left(ka\right)}\cos\left(n+\frac{1}{2}\right)\theta ,\;\;\;\;\;\;\;\;\;\;\;\;\;\;\;\;\;\;\;\;\;\;\;\;\;\;\;\;\theta \in \left(\theta_{0},\pi \right), \end{array}\right.$$

where C is an integration constant. A similar process applied to the dual series equations (6.4) and (6.5) results in


(6.11) $$\sum_{n=1}^{\infty }y_{n}\sin\left(n+\frac{1}{2}\right)\theta =\left\{ \begin{array}{l} -\frac{i}{ka}B_{1}\sin\frac{\theta }{2}+\sum_{n=1}^{\infty }y_{n}\mu_{n}\sin\left(n+\frac{1}{2}\right)\theta ,\;\;\theta \in \left(0,\theta_{0}\right)\;\\ iB_{3}\sin\frac{\theta }{2}+\sum_{n=1}^{\infty }\frac{i^{n}}{\varsigma_{n}\left(ka\right)}\sin\left(n+\frac{1}{2}\right)\theta ,\;\;\;\;\;\;\theta \in \left(\theta_{0},\pi \right). \end{array}\right.$$

The transformation of (6.10) and (6.11) (coupled via the constants $B_{1},B_{3}$) into infinite systems of linear algebraic equations is straightforward; in a similar way to the process used in §3, multiply both sides by $\cos\left(n+\frac{1}{2}\right)\theta$ or $\sin\left(n+\frac{1}{2}\right)\theta$ as appropriate and integrate over the complete interval. The definition of the constants $B_{1},B_{3}$ and C introduces no difficulty (see [5]). The result is a well-conditioned system of linear algebraic equations that is effectively and efficiently solved by the truncation method.

The rigorous solution of this basic problem gave rise to effective solutions for several other structures of practical interest in electro-engineering, including two concentric open spherical shells, and a solid or multi-layer dielectric sphere partially shielded by an open spherical cap [5]. Extremely large spherical reflector antennas were studied in [55], spanning low frequencies (λ≫a) to quasi-optics (λ≪a), where the wave size λ/a is measured in the thousands. The shielded constant K-Lens was examined in [56], and a shielded Luneberg lens in [57]. These solutions all possess the expected merits of the MAR: high quality (or benchmark) accuracy for evaluation of the results obtained with other numerical methods for which estimates of accuracy cannot be reliably given; and no limitations on the geometrical parameters or on the frequency band.

Apertures in the structures of the type shown in figure 4*b*,*c* require an additional tool. The formula (3.11) is employed for the solution of wave scattering problems from the PEC spherical shell with an equatorial slot (comprising two spherical caps), and the spherical barrel (possessing two circular apertures). The EM (and acoustic) wave scattering problems are rigorously solved in [58] and [59] (see also [4]). Although the deduction is too lengthy to present here, the essential ideas can be understood by considering the corresponding electrostatic problem (similarly to that done in §3). When one cap is charged to unit potential and the other cap to a potential of the same or opposite sign ${\left(-1\right)}^{l},$where l=0 or 1, the following symmetrical triple series equations involving Legendre polynomials $P_{n}\left(z\right)$ arise, where z$=\cos\vartheta ,z_{0}=\cos\vartheta_{0}$ :


(6.12) $$\left\{ \begin{array}{l} \sum_{n=0}^{\infty }x_{n}P_{n}\left(z\right)={\left(-1\right)}^{l},\;\;\;\;\;\;\;\;\;\;\;\;z\in \left(-1,-z_{0}\right)\\ \sum_{n=0}^{\infty }\left(n+\frac{1}{2}\right)x_{n}P_{n}\left(z\right)=0,\;\;\;\;\;z\in \left(-z_{0},z_{0}\right)\\ \sum_{n=0}^{\infty }x_{n}P_{n}\left(z\right)=1\;\;\;\;\;\;\;\;\;\;\;\;\;\;\;\;\;\;\;z\in \left(z_{0},1\right), \end{array}\right.$$

where ${\left\{x_{n}\right\}}_{0}^{\infty }$ denote the unknown coefficients to be determined; the condition of boundedness of the electrostatic energy W in any finite volume of space, including the sharp edges of the caps,


$$W=4\pi a\sum_{n=1}^{\infty }\frac{n}{2n+1}{\left|x_{n}\right|}^{2}<\infty ,$$

requires the coefficients ${\left\{x_{n}\right\}}_{0}^{\infty }$ to belong to $l_{2}$ (full details in [4]).

The symmetry of the Legendre polynomials $P_{n}\left(-z\right)={\left(-1\right)}^{n}P_{n}\left(z\right)$ allows (6.12) to be rewritten as two independent dual series equations for the odd and even index coefficients respectively, over the interval $\left(-1,0\right)$:


(6.13) $$\left\{ \begin{array}{ll} \sum_{n=0}^{\infty }x_{2n+l}P_{2n+l}(z)=(-1{)}^{l}, & z\in (-1,-z_{0})\\ \sum_{n=0}^{\infty }\left(n+\frac{l}{2}+\frac{1}{4}\right)x_{2n+l}(z)P_{2n+l}(z)=0, & z\in (-z_{0},0) \end{array}\right.\;\;\;\;\left(l=0,1\right).$$

Now using (3.12) and introducing the new variable $u=2z^{2}-1=\cos2\vartheta$, with $u_{0}=2z_{0}^{2}-1=\cos2\vartheta_{0}$, the equations (6.13) are transformed to the following dual series equations, defined on the interval $\left(-1,1\right)$:


(6.14) $$\left\{ \begin{array}{l} \sum_{n=0}^{\infty }\left(n+\frac{l}{2}+\frac{1}{4}\right)x_{2n+l}P_{n}^{\left(0,l-\frac{1}{2}\right)}\left(u\right)=0,\;\;\;\;\;\;\;\;\;\;\;\;\;\;\;\;\;\;\;\;\;\;u\in \left(-1,u_{0}\right)\\ \sum_{n=0}^{\infty }x_{2n+l}P_{n}^{\left(0,l-\frac{1}{2}\right)}\left(u\right)={\left(-1\right)}^{l}{\left\{\frac{1}{2}\left(1+u\right)\right\}}^{-\frac{1}{2}},\;\;\;\;\;\;u\in \left(u_{0},1\right). \end{array}\right.$$

The seemingly formal transition from equations (6.13) to (6.14) makes a highly significant change in the effectiveness of the final solution. Any direct solution of (6.13) is heavily dependent on the angular size $\vartheta_{0}$ of the charged spherical caps whereas the solution of (6.14) is uniformly valid with respect to $\vartheta_{0}$. To illustrate the Abel integral transform method for this type of system, we solve the even index (l=0) system:


(6.15) $$\left\{ \begin{array}{l} \sum_{n=0}^{\infty }\left(n+\frac{1}{4}\right)x_{2n}P_{n}^{\left(0,-\frac{1}{2}\right)}\left(u\right)=0,\;\;\;\;\;\;\;\;\;\;\;\;\;\;\;\;\;\;\;\;\;\;\;\;\;\;u\in \left(-1,u_{0}\right)\\ \sum_{n=0}^{\infty }x_{2n}P_{n}^{\left(0,-\frac{1}{2}\right)}\left(u\right)={\left\{\frac{1}{2}\left(1+u\right)\right\}}^{-\frac{1}{2}},\;\;\;\;\;\;\;\;\;\;\;\;\;\;\;\;\;\;u\in \left(u_{0},1\right). \end{array}\right.$$

First, we apply the direct Abel integral transform to the first equation in (6.15), using (3.7) with $\alpha =-\frac{1}{2}$, β=0,


$$\int_{-1}^{t}\frac{{\left(1+u\right)}^{-\frac{1}{2}}P_{n}^{\left(0,-\frac{1}{2}\right)}\left(u\right)}{{\left(t-u\right)}^{\frac{1}{2}}}du=\sqrt{\pi }\frac{\Gamma \left(n+\frac{1}{2}\right)}{\Gamma \left(n+1\right)}P_{n}^{\left(-\frac{1}{2},0\right)}\left(t\right),$$

to obtain


(6.16) $$\sum_{n=0}^{\infty }\left(n+\frac{1}{4}\right)\frac{\Gamma \left(n+\frac{1}{2}\right)}{\Gamma \left(n+1\right)}x_{2n}P_{n}^{\left(-\frac{1}{2},0\right)}\left(u\right)=0,\;\;\;\;\;\;\;\;\;u\in \left(-1,u_{0}\right).$$

The second equation in (6.15) is transformed into Abel’s IE using the representation (3.6),


$$P_{n}^{\left(0,-\frac{1}{2}\right)}\left(u\right)=\frac{\Gamma \left(n+1\right)}{\sqrt{\pi }\Gamma \left(n+\frac{1}{2}\right)}\int_{u}^{1}\frac{{\left(1-x\right)}^{-\frac{1}{2}}P_{n}^{\left(-\frac{1}{2},0\right)}\left(x\right)}{{\left(x-u\right)}^{\frac{1}{2}}}dx,$$

to arrive at the series equation


(6.17) $$\sum_{n=0}^{\infty }\frac{\Gamma \left(n+1\right)}{\Gamma \left(n+\frac{1}{2}\right)}x_{2n}P_{0}^{\left(-\frac{1}{2},0\right)}\left(u\right)=\frac{2\sqrt{2}}{\sqrt{\pi }},\;\;\;\;\;\;\;\;\;\;\;\;u\in \left(u_{0},1\right).$$

Rearrangement of (6.16) and (6.17) produces the desired format of equation (4.5):


(6.18) $$\sum_{n=0}^{\infty }c_{2n}P_{n}^{\left(-\frac{1}{2},0\right)}\left(u\right)=\left\{ \begin{array}{l} \sum_{n=0}^{\infty }c_{2n}\varepsilon_{n}^{\left(0\right)}c_{2n}P_{n}^{\left(-\frac{1}{2},0\right)}\left(u\right),\;\;\;u\in \left(-1,u_{0}\right)\\ \frac{2\sqrt{2}}{\sqrt{\pi }},\;\;\;\;\;\;\;\;\;\;\;\;\;\;\;\;\;\;\;\;\;\;\;\;\;\;\;\;\;\;\;\;\;\;\;\;\;u\in \left(u_{0},1\right), \end{array}\right.$$

where

,$$c_{2n}=\frac{\Gamma (n+1)}{\Gamma \left(n+\frac{1}{2}\right)}x_{2n},\;\;\varepsilon_{n}^{\left(0\right)}=1-\left(n+\frac{1}{4}\right){\left\{\frac{\Gamma \left(n+\frac{1}{2}\right)}{\left(n+1\right)}\right\}}^{2}$$

$(\varepsilon_{n}^{\left(0\right)}=O\left(n^{-2}\right)$ as n→∞). By employing the orthogonal properties of the Jacobi polynomials equation (6.18) is readily converted into a well-conditioned Fredholm matrix system of second kind, thus ensuring accurate solutions for the coefficients may be found by the truncation method. A similar process may be employed for the odd index coefficients. The calculation of the potential field (near- and far-field) is straightforward. Further details of the solution and analysis of this and other similar problems can be found in [4].

The approach to wave scattering problems utilizing Abel’s integral transform is nearly the same as the electrostatic case. The main difference is the addition to the equations of asymptotically small parameters of type (6.1) that provide a perturbation to the basic procedure, as demonstrated by the wave scattering problems presented in [5], and in the literature [60,61].

The next stage is to consider acoustic and electromagnetic wave scattering problems for open axisymmetric shells formed by the rotation of an arbitrary smooth generatrix about an axis. Historically, the MAR for two-dimensional wave scattering problems began with slotted circular cylinders [9], progressing to elliptic cylinders [36], and finally to cylinders of arbitrary profile [34]. A similar evolution occurred in the study of open axisymmetric shells. The first rigorous solution for a spherical shell with a single circular aperture [53,54], was followed by solutions (see [58,59]) for a spherical shell with an equatorial slot and a barrel-like open shell (a spherical shell with two symmetrical circular apertures). Seeking the extension to a wider class of rotationally symmetric shells, spheroidal shells present a natural first choice. Paralleling the paper [36] on a slotted elliptic cylinder, considerable additional complexity in the extraction of the singular part of the problem operator was incurred, not only from the radial spheroidal functions $R_{mn}\left(c,\xi \right)$ but also the angular spheroidal functions $S_{mn}\left(c,\eta \right)$, because both sets of functions depend on the frequency parameter c. Nevertheless, this procedure was successfully performed: chapter 7 in [5] is devoted to the analysis of various acoustic and EM wave scattering problems for open prolate spheroidal shells.

Open shells formed by the rotation of a smooth arbitrary generatrix present the greatest level of complexity. The decomposition of the initial problem operator is more challenging; the Abel integral transform method, however, is not modified. An outline of the approach appeared in [62]. To date, the Dirichlet problems for the Laplace [63] and Helmholtz [64,65] equations, governing electrostatic problems and acoustic wave scattering problems for a rotationally symmetric shell S, have been rigorously solved by the Abel transform approach. A point p on S may be described by a pair of parameters $\left(\tau_{p},\varphi_{p}\right)$. The solution is sought in the form of a single-layer potential [37]. Satisfaction of the boundary conditions produces a surface integral equation to be solved for the single-layer density $j(\tau_{p},\varphi_{p})$. The first step is to express the single-layer density, the three-dimensional Green’s function and the illuminating field as Fourier series in the azimuthal variable φ. For each index m=0,±1,±2,…, this produces a first kind integral equation with a singular kernel $G^{m}(\tau_{p},\tau_{q})$ for the component $j^{m}(\tau_{p})$ of the single layer density. In many aspects the split of the kernel into singular and regular parts follows the two-dimensional Dirichlet boundary problem for arbitrarily shaped cylinders. In turn, each IE is reduced to dual series equations involving the associated Legendre polynomials $P_{n}^{m}\left(\cos\theta \right)$ where m is fixed; for each m, these are converted to a well-conditioned matrix system of second kind. The full solution is then assembled from the inverse Fourier transform of the azimuthal components.

Some extensions in the application of the MAR to wave scattering problems have built on this approach. Rotationally symmetric ensembles of otherwise arbitrarily shaped shells each with a single aperture were treated in [66]. On the other hand, the generalization of the spheroidal barrel study, to a doubly connected rotationally symmetric screen of arbitrary shape, was successfully tackled in [67].

## 7. Conclusion

The method reviewed in this paper utilizes the Abel integral transform as its principal tool in the regularization of the ill-posed integral equations that occur in EM wave scattering problems and upon discretization give rise to ill-conditioned systems of linear equations. The method may be regarded as one of the family of different but equivalent procedures that are classed under the name of the Method of Analytical Regularization. The ill-conditioning is particularly acute for structures such as cavity backed apertures. When the scattered field is represented as a single- or double-layer potential, the enforcement of boundary conditions produces first kind integral equations. It is usual to expand the unknown surface density, the Green’s function kernel of the IE and the excitation in terms of Fourier series (in two-dimensional applications) or associated Legendre polynomials (in three-dimensional applications). This leads directly to dual (or higher order) series equations expressed in terms of either the trigonometrical functions ${\left\{\cos n\phi \right\}}_{n=0}^{\infty }$, ${\left\{\sin n\phi \right\}}_{n=1}^{\infty }$ or the associated Legendre polynomials $P_{n}^{m}\left(\cos\theta \right)$. The ill-conditioning manifests itself in the different rates of decay of the general term in each member of the series equations. The application of an appropriate *Abel integral transform* to each member of the series equations—expressed in terms of a suitable Jacobi polynomial—adjusts the rates of decay of the general term in each to be asymptotically equal, and in this form the series equations are readily converted to a matrix system of equations that is of second kind. Such systems are ideal for the application of standard algorithms that produce numerical solutions which are reliable and of guaranteed accuracy.

The three different structures reviewed in this paper were carefully chosen to exemplify the application of the method to arbitrary two-dimensional PEC scatterers in both E- and H-polarization and to three-dimensional axisymmetric scatterers that are either singly connected (possessing one aperture) or doubly connected (possessing two apertures). Extensions to the basic problems were briefly described as well as prospects for further development.

Despite the apparent complexity of the solution, the outcome has the significant advantage of providing one of the very few EM algorithms (of some generality) that gives solutions of guaranteed prescribed accuracy. In common with other implementations of the MAR (such as the Riemann–Hilbert approach and the Regularizing Galerkin Technique), it meets the strict criteria laid out in the definitive paper of Hsiao & Kleinman [68] concerning error estimation in numerical solutions of the integral equations arising in electromagnetics.

## Data Availability

This article has no additional data.
